# Iodido{4-phenyl-1-[1-(1,3-thia­zol-2-yl-κ*N*)ethyl­idene]thio­semicarbazidato-κ^2^ *N*′,*S*}{4-phenyl-1-[1-(1,3-thia­zol-2-yl)ethyl­idene]thio­semicarbazide-κ*S*}mercury(II)

**DOI:** 10.1107/S160053681101974X

**Published:** 2011-05-28

**Authors:** Samuel S. R. Dasary, Sri Ranjini Arumugam, Hongtao Yu, Ramaiyer Venkatraman, Frank R. Fronczek

**Affiliations:** a Department of Chemistry and Biochemistry, Jackson State University, Jackson, MS 39217, USA; b Department of Chemistry, Louisiana State University, Baton Rouge, LA 70803, USA

## Abstract

In the title compound, [Hg(C_12_H_11_N_4_S_2_)I(C_12_H_12_N_4_S_2_)], the Hg atom is in a distorted square-pyramidal coordination, defined by the iodide ligand, by the S atom of the neutral ligand in the apical position, and by the N atom of the thia­zole ring, the thio­ureido N and the S atom of the deprotonated ligand. The deprotonated ligand intra­molecularly hydrogen bonds to the thia­zole ring N atom, while the deprotonated ligand forms an inter­molecular hydrogen bond to the thiol­ate S atom. The deprotonation of the tridentate ligand and its coordination to Hg *via* the S atom strikingly affects the C—S bond lengths. In the free ligand, the C—S bond distance is 1.685 (7) Å, whereas it is 1.749 (7) Å in the deprotonated ligand. Similarly, the Hg—S bond distance is slightly longer to the neutral ligand [2.6682 (18) Å] than to the deprotonated ligand [2.5202 (19) Å]. The Hg—I distance is 2.7505 (8) Å.

## Related literature

For general background to thio­semicarbazones and their Hg complexes, see: Akinchan *et al.* (2002 ▶); Ali & Livingstone (1974 ▶); Bermejo *et al.* (1999 ▶, 2003 ▶); Lobana *et al.* (1998 ▶); Venkatraman *et al.* (2009 ▶); Blanz & French (1968 ▶); Campbell (1975 ▶); Casas *et al.* (2000 ▶); Grecu & Neamtu (1967 ▶); Pellerito & Negy (2002 ▶); Raper (1985 ▶).
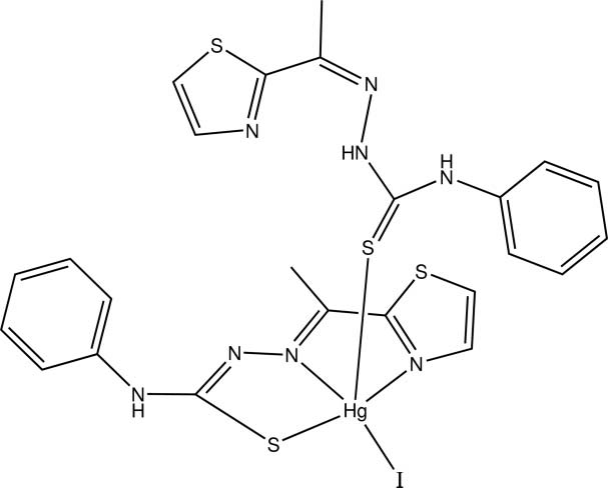


## Experimental

### 

#### Crystal data

[Hg(C_12_H_11_N_4_S_2_)I(C_12_H_12_N_4_S_2_)]
*M* *_r_* = 879.23
Triclinic, 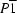

*a* = 8.694 (2) Å
*b* = 10.119 (2) Å
*c* = 16.801 (4) Å
α = 76.670 (13)°
β = 79.448 (12)°
γ = 77.190 (13)°
*V* = 1388.8 (5) Å^3^
*Z* = 2
Mo *K*α radiation
μ = 6.99 mm^−1^
*T* = 90 K
0.10 × 0.10 × 0.03 mm

#### Data collection

Nonius KappaCCD diffractometer with an Oxford Cryosystems Cryostream cooler
Absorption correction: multi-scan (*SCALEPACK*; Otwinowski & Minor, 1997 ▶) *T*_min_ = 0.542, *T*_max_ = 0.818
21861 measured reflections
5837 independent reflections
4289 reflections with *I* > 2σ(*I*)
*R*_int_ = 0.059

#### Refinement

*R*[*F* ^2^ > 2σ(*F* ^2^)] = 0.042
*wR*(*F* ^2^) = 0.085
*S* = 1.02
5837 reflections
346 parameters
H-atom parameters constrained
Δρ_max_ = 1.36 e Å^−3^
Δρ_min_ = −1.27 e Å^−3^

### 

Data collection: *COLLECT* (Nonius, 2000 ▶); cell refinement: *SCALEPACK* (Otwinowski & Minor, 1997 ▶); data reduction: *DENZO* (Otwinowski & Minor, 1997 ▶) and *SCALEPACK*; program(s) used to solve structure: *SIR97* (Altomare *et al.*, 1999 ▶); program(s) used to refine structure: *SHELXL97* (Sheldrick, 2008 ▶); molecular graphics: *ORTEP-3 for Windows* (Farrugia, 1997 ▶); software used to prepare material for publication: *SHELXL97*.

## Supplementary Material

Crystal structure: contains datablocks global, I. DOI: 10.1107/S160053681101974X/vm2089sup1.cif

Structure factors: contains datablocks I. DOI: 10.1107/S160053681101974X/vm2089Isup2.hkl

Additional supplementary materials:  crystallographic information; 3D view; checkCIF report

## Figures and Tables

**Table 1:** Hydrogen-bond geometry (Å, °)

*D*—H⋯*A*	*D*—H	H⋯*A*	*D*⋯*A*	*D*—H⋯*A*
N7—H7*N*⋯N5	0.88	1.97	2.667 (8)	135
N4—H4*N*⋯S2^i^	0.88	2.69	3.553 (6)	167

Symmetry code: (i) 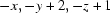.

**Fig. 1.**
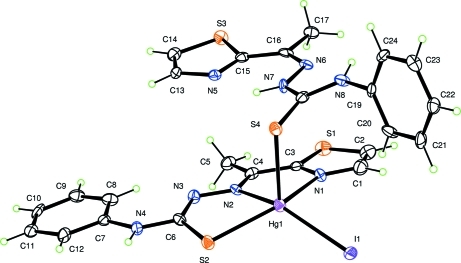
The molecular structure of the title compound. Displacement ellipsoids are drawn at the 50% probability level.

## supplementary crystallographic information

### Comment

Studies on thiosemicarbazones and their metal complexes has remained a fertile field of research for more than three decades due to their significant impacts in biology and chemistry (Ali & Livingstone, 1974; Campbell, 1975; Pellerito & Negy, 2002). Thiosemicarbazones are known to coordinate to many metals, potentially as tridentate ligands. The ligation can occur with the metals as a neutral molecule or, after deprotonation, as an anionic ligand (Campbell, 1975; Raper, 1985; Casas *et al.*, 2000). Further, the metal-chelating ability is attributed to the thione-thiol tautomerism exhibited by these molecules. Some of the metal complexes are found to exhibit enhanced biological activity compared to their basic ligand (Blanz & French, 1968). Among the metals studied with thiosemicarbazones, mercury and organomercury compounds are scarce (Grecu & Neamtu, 1967; Lobana *et al.*,1998; Bermejo *et al.*, 1999; Akinchan *et al.*, 2002; Bermejo *et al.*, 2003). Removal and remediation of many mercury species from environmental samples are very important. We report here the synthesis and structure of a mercury(II) iodide complex of the phenyl derivative of 2-acetylthiazole-3-thiosemicarbazone. The title complex is a result of interaction between two neutral ligand molecules and mercury(II) iodide in methanol. In this complex, Hg(II) is chelated by two 2-acetylthiazole-3-phenylthiosemicarbazone ligands, forming a distorted square pyramidal geometry (Fig 1). One of the two ligands is deprotonated at N3, and tridentate through its N, N, S atoms. Along with the iodide atom, it forms the square planar base. The other ligand is neutral and apical to the Hg atom, binding through its S atom. The proton NMR also provides the evidence of ligand deprotonation during metal chelation. The sharp resonance signal due to N—NH proton at 11.12 p.p.m. disappears in the spectrum of the complex. The N4 signal (at ~8.64 p.p.m.) in the ligand undergoes a downfield shift that is marked in the mercury (II) complexes, indicating coordination *via* the S atom. The protonated and deprotonated ligands have different conformations, differing primarily by the N–N–C–N torsion angle, which is *antiperiplanar* in the deprotonated ligand (torsion angle N2—N3—C6—N4 179.6 (5)°) and *synperiplanar* in the neutral ligand (torsion angle N6—N7—C18—N8 9.3 (9)°). Hydrogen bonding details are given in Table 1.

### Experimental

To a solution of 2-acetyl thiazole thiosemicarbazone (Venkatraman *et al.* 2009) (1.38 g, 5 mmol) in warm methanol (50 ml) was added an equimolar methanol solution (50 ml) of mercury(II) iodide (1.36 g. 5 mmol). The mixture was stirred for about 24 h, after which time the yellow solid obtained was filtered and vacuum dried (yield ~75%). Crystals suitable for diffraction studies were obtained from the mother liquor at room temperature after a week.

### Refinement

All H atoms were placed in calculated positions, guided by difference maps, with C—H bond distances 0.95–0.98 Å, N—H 0.88 Å, and thereafter refined as riding with *U*_iso_= *xU*_eq_, where *x* = 1.5 for methyl H and 1.2 for all other H atoms. The highest peak in the final difference map was 1.29 Å from the Hg position.

### Crystal data

**Table:**

[Hg(C_12_H_11_N_4_S_2_)I(C_12_H_12_N_4_S_2_)]	*Z* = 2
*M**_r_* = 879.23	*F*(000) = 840
Triclinic, *P*1	*D*_x_ = 2.103 Mg m^−^^3^
Hall symbol: -P 1	Mo *K*α radiation, λ = 0.71073 Å
*a* = 8.694 (2) Å	Cell parameters from 5532 reflections
*b* = 10.119 (2) Å	θ = 2.5–26.7°
*c* = 16.801 (4) Å	µ = 6.99 mm^−^^1^
α = 76.670 (13)°	*T* = 90 K
β = 79.448 (12)°	Parallelepiped, yellow
γ = 77.190 (13)°	0.10 × 0.10 × 0.03 mm
*V* = 1388.8 (5) Å^3^	

### Data collection

**Table:**

Nonius KappaCCD diffractometer with an Oxford Cryosystems Cryostream cooler	5837 independent reflections
Radiation source: fine-focus sealed tube	4289 reflections with *I* > 2σ(*I*)
graphite	*R*_int_ = 0.059
ω and φ scans	θ_max_ = 26.7°, θ_min_ = 2.8°
Absorption correction: multi-scan (*SCALEPACK*; Otwinowski & Minor, 1997)	*h* = −10→10
*T*_min_ = 0.542, *T*_max_ = 0.818	*k* = −11→12
21861 measured reflections	*l* = −21→21

### Refinement

**Table:**

Refinement on *F*^2^	Secondary atom site location: difference Fourier map
Least-squares matrix: full	Hydrogen site location: inferred from neighbouring sites
*R*[*F*^2^ > 2σ(*F*^2^)] = 0.042	H-atom parameters constrained
*wR*(*F*^2^) = 0.085	*w* = 1/[σ^2^(*F*_o_^2^) + (0.0336*P*)^2^] where *P* = (*F*_o_^2^ + 2*F*_c_^2^)/3
*S* = 1.02	(Δ/σ)_max_ = 0.001
5837 reflections	Δρ_max_ = 1.36 e Å^−^^3^
346 parameters	Δρ_min_ = −1.27 e Å^−^^3^
0 restraints	Extinction correction: *SHELXL97* (Sheldrick, 2008), Fc^*^=kFc[1+0.001xFc^2^λ^3^/sin(2θ)]^-1/4^
Primary atom site location: structure-invariant direct methods	Extinction coefficient: 0.00093 (17)

### Special details

**Table:**

Geometry. All e.s.d.'s (except the e.s.d. in the dihedral angle between two l.s. planes) are estimated using the full covariance matrix. The cell e.s.d.'s are taken into account individually in the estimation of e.s.d.'s in distances, angles and torsion angles; correlations between e.s.d.'s in cell parameters are only used when they are defined by crystal symmetry. An approximate (isotropic) treatment of cell e.s.d.'s is used for estimating e.s.d.'s involving l.s. planes.
Refinement. Refinement of *F*^2^ against ALL reflections. The weighted *R*-factor *wR* and goodness of fit *S* are based on *F*^2^, conventional *R*-factors *R* are based on *F*, with *F* set to zero for negative *F*^2^. The threshold expression of *F*^2^ > σ(*F*^2^) is used only for calculating *R*-factors(gt) *etc*. and is not relevant to the choice of reflections for refinement. *R*-factors based on *F*^2^ are statistically about twice as large as those based on *F*, and *R*- factors based on ALL data will be even larger.

### Fractional atomic coordinates and isotropic or equivalent isotropic displacement parameters (Å^2^)

**Table:**

	*x*	*y*	*z*	*U*_iso_*/*U*_eq_	
Hg1	0.31186 (3)	0.89942 (3)	0.265558 (18)	0.01642 (10)	
I1	0.21508 (5)	1.07878 (4)	0.12625 (3)	0.01901 (13)	
S1	0.8957 (2)	0.80421 (19)	0.20242 (13)	0.0233 (4)	
S2	0.1598 (2)	0.96404 (18)	0.39821 (11)	0.0190 (4)	
S3	0.9843 (2)	0.36980 (18)	0.41473 (12)	0.0203 (4)	
S4	0.2697 (2)	0.64537 (17)	0.26747 (11)	0.0160 (4)	
N1	0.5975 (7)	0.8585 (6)	0.1973 (4)	0.0172 (13)	
N2	0.5053 (6)	0.8499 (5)	0.3621 (4)	0.0140 (13)	
N3	0.4585 (6)	0.8448 (6)	0.4445 (4)	0.0167 (13)	
N4	0.2463 (7)	0.8923 (5)	0.5448 (4)	0.0167 (13)	
H4N	0.1422	0.9187	0.5536	0.020*	
N5	0.6878 (7)	0.4722 (5)	0.4101 (4)	0.0165 (13)	
N6	0.7246 (6)	0.4946 (5)	0.2273 (4)	0.0148 (13)	
N7	0.5745 (6)	0.5393 (5)	0.2660 (4)	0.0153 (13)	
H7N	0.5599	0.5437	0.3186	0.018*	
N8	0.4802 (7)	0.5470 (5)	0.1473 (4)	0.0193 (14)	
H8N	0.5761	0.5022	0.1321	0.023*	
C1	0.6692 (9)	0.8629 (7)	0.1192 (5)	0.0246 (18)	
H1	0.6108	0.8833	0.0739	0.029*	
C2	0.8316 (9)	0.8360 (7)	0.1094 (5)	0.0263 (19)	
H2	0.8988	0.8352	0.0580	0.032*	
C3	0.6998 (8)	0.8271 (6)	0.2493 (5)	0.0176 (16)	
C4	0.6563 (8)	0.8224 (7)	0.3384 (4)	0.0162 (16)	
C5	0.7824 (8)	0.7881 (7)	0.3942 (5)	0.0220 (17)	
H5A	0.7641	0.8587	0.4280	0.033*	
H5B	0.8875	0.7858	0.3608	0.033*	
H5C	0.7778	0.6975	0.4303	0.033*	
C6	0.3053 (9)	0.8937 (7)	0.4635 (4)	0.0174 (16)	
C7	0.3180 (8)	0.8573 (6)	0.6171 (4)	0.0159 (16)	
C8	0.4837 (8)	0.8114 (7)	0.6195 (5)	0.0199 (17)	
H8	0.5544	0.7976	0.5706	0.024*	
C9	0.5407 (9)	0.7870 (7)	0.6942 (5)	0.0216 (17)	
H9	0.6518	0.7563	0.6957	0.026*	
C10	0.4416 (9)	0.8057 (7)	0.7667 (5)	0.0222 (17)	
H10	0.4838	0.7899	0.8171	0.027*	
C11	0.2783 (9)	0.8484 (7)	0.7643 (5)	0.0194 (16)	
H11	0.2079	0.8606	0.8135	0.023*	
C12	0.2183 (9)	0.8730 (7)	0.6898 (4)	0.0198 (17)	
H12	0.1068	0.9012	0.6889	0.024*	
C13	0.7128 (8)	0.4433 (6)	0.4910 (4)	0.0153 (15)	
H13	0.6288	0.4601	0.5345	0.018*	
C14	0.8635 (8)	0.3898 (7)	0.5051 (5)	0.0206 (17)	
H14	0.8980	0.3663	0.5579	0.025*	
C15	0.8228 (8)	0.4387 (6)	0.3616 (4)	0.0159 (16)	
C16	0.8390 (8)	0.4512 (6)	0.2728 (5)	0.0165 (16)	
C17	1.0026 (8)	0.4099 (7)	0.2277 (5)	0.0194 (17)	
H17A	0.9990	0.4341	0.1680	0.029*	
H17B	1.0388	0.3099	0.2441	0.029*	
H17C	1.0766	0.4587	0.2415	0.029*	
C18	0.4504 (8)	0.5761 (7)	0.2232 (4)	0.0164 (16)	
C19	0.3674 (8)	0.5833 (7)	0.0882 (4)	0.0151 (15)	
C20	0.3171 (8)	0.7201 (7)	0.0526 (5)	0.0209 (17)	
H20	0.3519	0.7919	0.0682	0.025*	
C21	0.2147 (8)	0.7508 (8)	−0.0067 (4)	0.0213 (17)	
H21	0.1763	0.8444	−0.0305	0.026*	
C22	0.1693 (8)	0.6466 (7)	−0.0307 (4)	0.0195 (17)	
H22	0.1029	0.6680	−0.0728	0.023*	
C23	0.2196 (8)	0.5104 (7)	0.0058 (4)	0.0176 (16)	
H23	0.1845	0.4386	−0.0096	0.021*	
C24	0.3209 (8)	0.4785 (7)	0.0647 (4)	0.0151 (15)	
H24	0.3581	0.3848	0.0889	0.018*	

### Atomic displacement parameters (Å^2^)

**Table:**

	*U*^11^	*U*^22^	*U*^33^	*U*^12^	*U*^13^	*U*^23^
Hg1	0.01578 (16)	0.01912 (16)	0.01491 (17)	−0.00140 (11)	−0.00418 (11)	−0.00464 (11)
I1	0.0239 (3)	0.0162 (2)	0.0163 (3)	−0.0001 (2)	−0.0067 (2)	−0.0026 (2)
S1	0.0140 (10)	0.0241 (10)	0.0307 (12)	−0.0032 (8)	−0.0002 (8)	−0.0058 (9)
S2	0.0186 (10)	0.0227 (9)	0.0150 (10)	0.0023 (8)	−0.0049 (8)	−0.0061 (8)
S3	0.0177 (10)	0.0235 (9)	0.0218 (11)	−0.0023 (8)	−0.0075 (8)	−0.0061 (8)
S4	0.0152 (9)	0.0190 (9)	0.0153 (10)	−0.0037 (8)	−0.0029 (7)	−0.0049 (7)
N1	0.020 (3)	0.019 (3)	0.014 (4)	−0.003 (3)	−0.001 (3)	−0.006 (3)
N2	0.015 (3)	0.013 (3)	0.015 (3)	−0.002 (2)	−0.001 (3)	−0.007 (2)
N3	0.013 (3)	0.020 (3)	0.015 (3)	−0.001 (3)	−0.001 (3)	−0.003 (3)
N4	0.016 (3)	0.020 (3)	0.013 (3)	0.000 (3)	−0.002 (3)	−0.003 (3)
N5	0.020 (3)	0.012 (3)	0.018 (4)	−0.004 (3)	0.000 (3)	−0.003 (3)
N6	0.012 (3)	0.011 (3)	0.021 (4)	−0.004 (2)	0.005 (3)	−0.007 (3)
N7	0.019 (3)	0.020 (3)	0.007 (3)	−0.003 (3)	−0.004 (3)	−0.003 (2)
N8	0.020 (3)	0.015 (3)	0.024 (4)	0.003 (3)	−0.008 (3)	−0.009 (3)
C1	0.029 (5)	0.024 (4)	0.021 (5)	−0.007 (4)	−0.007 (4)	−0.002 (3)
C2	0.026 (4)	0.023 (4)	0.028 (5)	−0.003 (4)	−0.005 (4)	−0.002 (4)
C3	0.020 (4)	0.009 (3)	0.020 (4)	−0.001 (3)	0.004 (3)	−0.001 (3)
C4	0.016 (4)	0.015 (3)	0.020 (4)	−0.002 (3)	−0.010 (3)	−0.002 (3)
C5	0.024 (4)	0.020 (4)	0.023 (4)	−0.003 (3)	−0.011 (3)	−0.002 (3)
C6	0.028 (4)	0.014 (3)	0.015 (4)	−0.007 (3)	−0.005 (3)	−0.008 (3)
C7	0.024 (4)	0.011 (3)	0.014 (4)	−0.004 (3)	−0.007 (3)	−0.003 (3)
C8	0.016 (4)	0.018 (4)	0.027 (5)	−0.001 (3)	−0.007 (3)	−0.006 (3)
C9	0.019 (4)	0.019 (4)	0.027 (5)	0.002 (3)	−0.008 (3)	−0.005 (3)
C10	0.041 (5)	0.016 (4)	0.013 (4)	−0.014 (4)	−0.012 (4)	0.006 (3)
C11	0.030 (4)	0.015 (3)	0.017 (4)	−0.010 (3)	−0.006 (3)	−0.004 (3)
C12	0.027 (4)	0.022 (4)	0.014 (4)	−0.005 (3)	−0.011 (3)	−0.002 (3)
C13	0.019 (4)	0.013 (3)	0.012 (4)	−0.005 (3)	0.000 (3)	−0.001 (3)
C14	0.023 (4)	0.019 (4)	0.020 (4)	−0.005 (3)	−0.004 (3)	−0.001 (3)
C15	0.021 (4)	0.010 (3)	0.020 (4)	−0.004 (3)	−0.008 (3)	−0.003 (3)
C16	0.021 (4)	0.010 (3)	0.023 (4)	−0.003 (3)	−0.006 (3)	−0.008 (3)
C17	0.018 (4)	0.017 (4)	0.025 (5)	−0.004 (3)	−0.004 (3)	−0.006 (3)
C18	0.025 (4)	0.011 (3)	0.016 (4)	−0.007 (3)	−0.010 (3)	−0.001 (3)
C19	0.014 (4)	0.020 (4)	0.007 (4)	0.001 (3)	−0.001 (3)	0.000 (3)
C20	0.023 (4)	0.019 (4)	0.025 (5)	0.000 (3)	−0.006 (3)	−0.014 (3)
C21	0.022 (4)	0.029 (4)	0.011 (4)	0.000 (3)	−0.007 (3)	−0.002 (3)
C22	0.022 (4)	0.023 (4)	0.015 (4)	−0.006 (3)	−0.005 (3)	−0.003 (3)
C23	0.015 (4)	0.025 (4)	0.020 (4)	−0.010 (3)	−0.005 (3)	−0.011 (3)
C24	0.014 (4)	0.012 (3)	0.015 (4)	−0.002 (3)	−0.001 (3)	0.003 (3)

### Geometric parameters (Å, °)

**Table:**

Hg1—N2	2.441 (6)	C5—H5A	0.9800
Hg1—S2	2.5202 (19)	C5—H5B	0.9800
Hg1—N1	2.525 (6)	C5—H5C	0.9800
Hg1—S4	2.6682 (18)	C7—C12	1.382 (10)
Hg1—I1	2.7505 (8)	C7—C8	1.417 (9)
S1—C2	1.693 (8)	C8—C9	1.383 (10)
S1—C3	1.730 (7)	C8—H8	0.9500
S2—C6	1.749 (7)	C9—C10	1.383 (10)
S3—C14	1.706 (8)	C9—H9	0.9500
S3—C15	1.730 (7)	C10—C11	1.394 (10)
S4—C18	1.685 (7)	C10—H10	0.9500
N1—C3	1.299 (9)	C11—C12	1.392 (9)
N1—C1	1.341 (9)	C11—H11	0.9500
N2—C4	1.287 (8)	C12—H12	0.9500
N2—N3	1.361 (8)	C13—C14	1.344 (9)
N3—C6	1.321 (9)	C13—H13	0.9500
N4—C6	1.366 (9)	C14—H14	0.9500
N4—C7	1.403 (8)	C15—C16	1.450 (10)
N4—H4N	0.8800	C16—C17	1.503 (9)
N5—C15	1.326 (9)	C17—H17A	0.9800
N5—C13	1.370 (9)	C17—H17B	0.9800
N6—C16	1.304 (8)	C17—H17C	0.9800
N6—N7	1.376 (7)	C19—C24	1.372 (9)
N7—C18	1.342 (8)	C19—C20	1.382 (9)
N7—H7N	0.8800	C20—C21	1.393 (9)
N8—C18	1.340 (9)	C20—H20	0.9500
N8—C19	1.454 (8)	C21—C22	1.367 (10)
N8—H8N	0.8800	C21—H21	0.9500
C1—C2	1.364 (10)	C22—C23	1.382 (9)
C1—H1	0.9500	C22—H22	0.9500
C2—H2	0.9500	C23—C24	1.380 (9)
C3—C4	1.468 (10)	C23—H23	0.9500
C4—C5	1.503 (9)	C24—H24	0.9500
			
N2—Hg1—S2	73.61 (13)	N4—C7—C8	124.5 (6)
N2—Hg1—N1	66.52 (19)	C9—C8—C7	118.9 (7)
S2—Hg1—N1	137.91 (14)	C9—C8—H8	120.5
N2—Hg1—S4	101.14 (13)	C7—C8—H8	120.5
S2—Hg1—S4	106.40 (6)	C8—C9—C10	122.3 (7)
N1—Hg1—S4	94.22 (13)	C8—C9—H9	118.8
N2—Hg1—I1	143.13 (13)	C10—C9—H9	118.8
S2—Hg1—I1	113.54 (4)	C9—C10—C11	118.6 (7)
N1—Hg1—I1	91.86 (13)	C9—C10—H10	120.7
S4—Hg1—I1	110.31 (4)	C11—C10—H10	120.7
C2—S1—C3	89.6 (4)	C12—C11—C10	120.1 (7)
C6—S2—Hg1	99.8 (2)	C12—C11—H11	120.0
C14—S3—C15	89.6 (4)	C10—C11—H11	120.0
C18—S4—Hg1	101.0 (2)	C7—C12—C11	121.3 (7)
C3—N1—C1	112.0 (6)	C7—C12—H12	119.4
C3—N1—Hg1	113.2 (5)	C11—C12—H12	119.4
C1—N1—Hg1	134.8 (5)	C14—C13—N5	115.8 (6)
C4—N2—N3	116.5 (6)	C14—C13—H13	122.1
C4—N2—Hg1	121.9 (5)	N5—C13—H13	122.1
N3—N2—Hg1	121.5 (4)	C13—C14—S3	110.5 (6)
C6—N3—N2	113.7 (6)	C13—C14—H14	124.7
C6—N4—C7	133.0 (6)	S3—C14—H14	124.7
C6—N4—H4N	113.5	N5—C15—C16	125.3 (6)
C7—N4—H4N	113.5	N5—C15—S3	113.5 (5)
C15—N5—C13	110.6 (6)	C16—C15—S3	121.1 (5)
C16—N6—N7	117.3 (6)	N6—C16—C15	126.4 (6)
C18—N7—N6	119.7 (6)	N6—C16—C17	115.7 (6)
C18—N7—H7N	120.1	C15—C16—C17	117.9 (6)
N6—N7—H7N	120.1	C16—C17—H17A	109.5
C18—N8—C19	125.5 (6)	C16—C17—H17B	109.5
C18—N8—H8N	117.2	H17A—C17—H17B	109.5
C19—N8—H8N	117.2	C16—C17—H17C	109.5
N1—C1—C2	115.3 (7)	H17A—C17—H17C	109.5
N1—C1—H1	122.3	H17B—C17—H17C	109.5
C2—C1—H1	122.3	N8—C18—N7	115.6 (6)
C1—C2—S1	109.8 (6)	N8—C18—S4	124.3 (5)
C1—C2—H2	125.1	N7—C18—S4	120.0 (5)
S1—C2—H2	125.1	C24—C19—C20	121.1 (6)
N1—C3—C4	124.1 (6)	C24—C19—N8	118.4 (6)
N1—C3—S1	113.3 (5)	C20—C19—N8	120.3 (6)
C4—C3—S1	122.5 (6)	C19—C20—C21	118.8 (7)
N2—C4—C3	114.2 (6)	C19—C20—H20	120.6
N2—C4—C5	125.1 (7)	C21—C20—H20	120.6
C3—C4—C5	120.7 (6)	C22—C21—C20	120.2 (7)
C4—C5—H5A	109.5	C22—C21—H21	119.9
C4—C5—H5B	109.5	C20—C21—H21	119.9
H5A—C5—H5B	109.5	C21—C22—C23	120.3 (7)
C4—C5—H5C	109.5	C21—C22—H22	119.8
H5A—C5—H5C	109.5	C23—C22—H22	119.8
H5B—C5—H5C	109.5	C24—C23—C22	120.1 (6)
N3—C6—N4	118.2 (6)	C24—C23—H23	120.0
N3—C6—S2	129.1 (6)	C22—C23—H23	120.0
N4—C6—S2	112.7 (5)	C19—C24—C23	119.4 (6)
C12—C7—N4	116.6 (6)	C19—C24—H24	120.3
C12—C7—C8	118.9 (7)	C23—C24—H24	120.3
			
N2—Hg1—S2—C6	−10.2 (3)	N2—N3—C6—N4	179.6 (5)
N1—Hg1—S2—C6	−29.3 (3)	N2—N3—C6—S2	−0.8 (9)
S4—Hg1—S2—C6	86.9 (2)	C7—N4—C6—N3	5.6 (11)
I1—Hg1—S2—C6	−151.6 (2)	C7—N4—C6—S2	−174.0 (6)
N2—Hg1—S4—C18	−66.7 (3)	Hg1—S2—C6—N3	11.2 (7)
S2—Hg1—S4—C18	−142.7 (3)	Hg1—S2—C6—N4	−169.2 (4)
N1—Hg1—S4—C18	0.1 (3)	C6—N4—C7—C12	178.0 (7)
I1—Hg1—S4—C18	93.7 (3)	C6—N4—C7—C8	−0.1 (11)
N2—Hg1—N1—C3	2.4 (4)	C12—C7—C8—C9	−1.5 (10)
S2—Hg1—N1—C3	22.4 (6)	N4—C7—C8—C9	176.6 (6)
S4—Hg1—N1—C3	−97.9 (5)	C7—C8—C9—C10	0.0 (10)
I1—Hg1—N1—C3	151.6 (4)	C8—C9—C10—C11	1.2 (10)
N2—Hg1—N1—C1	−176.8 (7)	C9—C10—C11—C12	−0.9 (10)
S2—Hg1—N1—C1	−156.8 (5)	N4—C7—C12—C11	−176.5 (6)
S4—Hg1—N1—C1	82.9 (6)	C8—C7—C12—C11	1.8 (10)
I1—Hg1—N1—C1	−27.6 (6)	C10—C11—C12—C7	−0.6 (10)
S2—Hg1—N2—C4	−168.3 (5)	C15—N5—C13—C14	0.5 (8)
N1—Hg1—N2—C4	−2.1 (5)	N5—C13—C14—S3	−1.0 (8)
S4—Hg1—N2—C4	87.7 (5)	C15—S3—C14—C13	1.0 (5)
I1—Hg1—N2—C4	−60.8 (6)	C13—N5—C15—C16	177.9 (6)
S2—Hg1—N2—N3	14.3 (4)	C13—N5—C15—S3	0.3 (7)
N1—Hg1—N2—N3	−179.5 (5)	C14—S3—C15—N5	−0.7 (5)
S4—Hg1—N2—N3	−89.7 (4)	C14—S3—C15—C16	−178.4 (6)
I1—Hg1—N2—N3	121.8 (4)	N7—N6—C16—C15	3.3 (10)
C4—N2—N3—C6	170.4 (6)	N7—N6—C16—C17	−177.1 (5)
Hg1—N2—N3—C6	−12.1 (7)	N5—C15—C16—N6	−1.2 (11)
C16—N6—N7—C18	−175.4 (6)	S3—C15—C16—N6	176.2 (5)
C3—N1—C1—C2	−0.8 (9)	N5—C15—C16—C17	179.2 (6)
Hg1—N1—C1—C2	178.4 (5)	S3—C15—C16—C17	−3.4 (8)
N1—C1—C2—S1	−0.1 (8)	C19—N8—C18—N7	−176.6 (6)
C3—S1—C2—C1	0.6 (6)	C19—N8—C18—S4	6.9 (10)
C1—N1—C3—C4	176.5 (6)	N6—N7—C18—N8	9.3 (9)
Hg1—N1—C3—C4	−2.9 (8)	N6—N7—C18—S4	−174.1 (4)
C1—N1—C3—S1	1.2 (7)	Hg1—S4—C18—N8	−109.2 (6)
Hg1—N1—C3—S1	−178.1 (3)	Hg1—S4—C18—N7	74.5 (5)
C2—S1—C3—N1	−1.1 (5)	C18—N8—C19—C24	−116.1 (7)
C2—S1—C3—C4	−176.4 (6)	C18—N8—C19—C20	68.1 (9)
N3—N2—C4—C3	179.0 (5)	C24—C19—C20—C21	1.5 (11)
Hg1—N2—C4—C3	1.5 (8)	N8—C19—C20—C21	177.1 (6)
N3—N2—C4—C5	−1.2 (9)	C19—C20—C21—C22	−2.1 (11)
Hg1—N2—C4—C5	−178.7 (5)	C20—C21—C22—C23	2.5 (11)
N1—C3—C4—N2	1.2 (10)	C21—C22—C23—C24	−2.4 (11)
S1—C3—C4—N2	175.9 (5)	C20—C19—C24—C23	−1.3 (10)
N1—C3—C4—C5	−178.7 (6)	N8—C19—C24—C23	−177.0 (6)
S1—C3—C4—C5	−3.9 (9)	C22—C23—C24—C19	1.8 (10)

### Hydrogen-bond geometry (Å, °)

**Table:**

*D*—H···*A*	*D*—H	H···*A*	*D*···*A*	*D*—H···*A*
N7—H7N···N5	0.88	1.97	2.667 (8)	135
N4—H4N···S2^i^	0.88	2.69	3.553 (6)	167

Symmetry codes: (i) −*x*, −*y*+2, −*z*+1.
